# Acute Intestinal Obstruction: A Rare Aetiology

**DOI:** 10.1155/2012/501209

**Published:** 2012-06-26

**Authors:** Shamita Chatterjee, Souvik Chatterjee, Sanjeev Kumar, Shahana Gupta

**Affiliations:** Department of Surgery, Medical College, Kolkata-700026, India

## Abstract

Internal herniation of small intestine is a very rare entity, and it poses a real diagnostic challenge clinically. Recurrent entrapment of the bowel may lead to partial to complete intestinal obstruction and eventually strangulation of the small bowel. Of this rare clinical entity, left paraduodenal hernia is more common. High index of suspicion with prompt management may prevent bowel strangulation and gangrene. We present a case of acute intestinal obstruction due to left paraduodenal hernia with malrotation of midgut in a 55-year-old male patient.

## 1. Introduction 

Internal herniation, either congenital or acquired, is a rare cause of dynamic intestinal obstruction. Paraduodenal hernias, which are a type of internal hernia, occur due to malrotation of midgut in peritoneal spaces and folds near the ligament of Treitz. Most of the internal hernias are incidentally detected during laparotomy or autopsy. Sometimes, they may present with the symptoms of intestinal obstruction with its dreaded complications like strangulation and gangrene. A high index of suspicion and prompt surgical intervention is required to minimize the morbidity and mortality. 

## 2. Case Report

A 55-year-old male patient presented to our emergency with abdominal pain, distension, and absolute constipation for two days. The patient had three similar episodes in the past, which responded to conservative management. There was no history of abdominal surgery or trauma. The abdominal examination revealed distension with rebound tenderness and hurried intestinal peristaltic sounds. On digital rectal examination, rectum was empty with ballooning. 

Hematological investigations demonstrated neutrophilic leucocytosis. Abdominal X-ray on erect posture revealed multiple air fluid levels with presence of valvulae conniventes. Ultrasonography demonstrated dilated bowel loops without any ascites.

**Figure 1:**
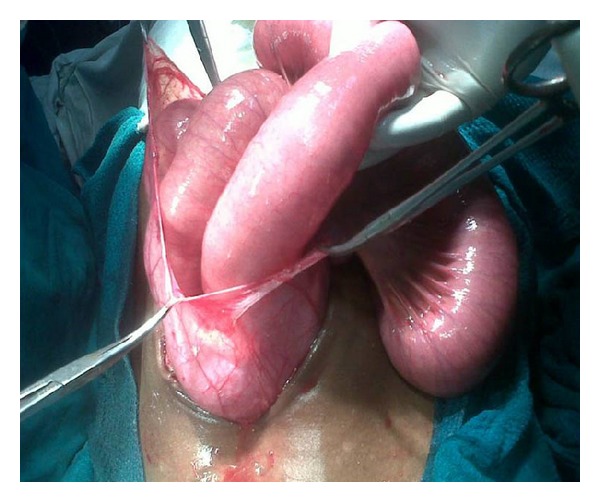
Entire small gut in the left paraduodenal area enclosed in a hernia sac.

**Figure 2:**
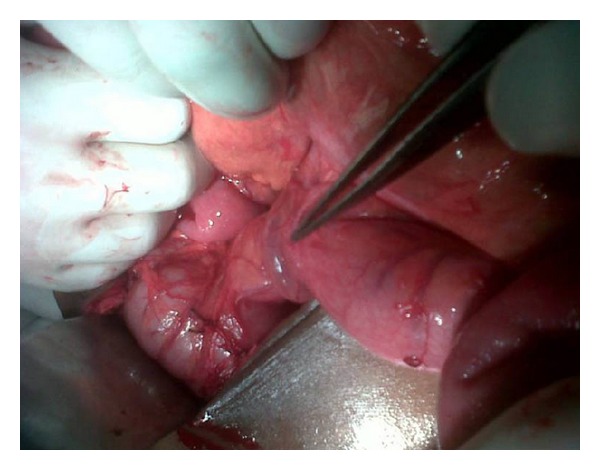
Inferior mesenteric vein situated on the right free margin of hernia sac.

The patient underwent exploratory laparotomy for acute intestinal obstruction. On exploration, almost entire small gut was found to be in the left paraduodenal area enclosed in a hernial sac (Figure 1). The inferior mesenteric vein was situated on the right free margin of hernia sac and at the region of ileocecal junction, it passed onto the anterior aspect of terminal ileum (Figure 2). The small gut, within the sac, was distended and congested. The peritoneal attachment along the right free margin of inferior mesenteric vein was excised, and the herniated small gut was reduced beneath the inferior mesenteric vein to its normal position on the left side of the base of the mesentery of small intestine. The peritoneal attachment at the neck of the hernia sac was sutured with the retroperitoneum; thus closing the neck of the hernial orifice. The postoperative recovery was uneventful. On followup, for next one year, the patient has been free from any further abdominal problems. 

## 3. Discussion 

Internal hernia is a rare phenomenon having an incidence of 1-2% [1, 2]. It may occur due to abnormal herniation of a viscus, usually small gut, through a normal (e.g., foramen of Winslow) or an abnormal opening (paraduodenal, ileocaecal, and sigmoid mesocolic hernia) within the peritoneal cavity. The abnormal opening may either be congenital or acquired. The congenital variety may be associated with malrotation of gut, while the acquired variety may occur following trauma, operation, inflammation, and so forth. Of this rare phenomenon of internal herniation, paraduodenal hernia is more common [1, 2]. Paraduodenal hernias of the left side are again more common than those of the right, though both have different embryological developments. Left paraduodenal hernia occurs when bowel protrudes through the fossa of Landzert due to elevation of a fold of peritoneum (plica venosa) by the inferior mesenteric vein [3]. As the herniation occurs though the unsupported area of descending mesocolon between inferior mesenteric vein and posterior parietal attachments, paraduodenal hernia is often considered a misnomer and is referred to as a mesocolic hernia [4]. 

The inferior mesenteric vein lies on the anterior free margin of the hernial sac [5]. It is believed that either there is reversed rotation of midgut into mesocolon or there is herniation of midgut loops through a congenital or pathological defect between posterior parities and the inferior mesenteric vein just below the fourth part of duodenum. In our patient, almost the whole length of the small intestine was encased by the layer of peritoneum. In the peritoneal sac, the small bowel remains in a clustered manner causing compression and obstruction of bowel as well as blood vessels [6].

Preoperative diagnosis of paraduodenal hernia possesses a real clinical challenge due its nonspecific symptoms. Sometimes, the patient may give a history of chronic postprandial pain for many years. But, it may present as acute intestinal obstruction, bowel ischemia, gangrene, even perforation of bowel [7]. The findings of plain radiography and barium follow through study are nonspecific. Abdominal CT scan demonstrates “a bag of bowel” in the hernial sac [8]. The timing of the imaging is very important, as the herniation is often intermittent and may not be picked up during imaging, if not done during symptomatic period. 

The left paraduodenal hernia is treated with excision of peritoneal sac, normal repositioning of the small gut, and suturing of the neck of the peritoneal sac with retroperitoneum. Care has to be taken not to injure the inferior mesenteric vein, while dissecting the peritoneal sac. Our patient is now free of any abdominal symptoms for the last one year following his treatment. 

## 4. Conclusion 

Internal herniation, if not diagnosed properly and in time bears the risk of ischemia, gangrene, even perforation leading to morbidity and even mortality. Therefore thorough knowledge, high index of clinical suspicion and immediate surgical intervention is essential to reduce the morbidity and mortality in such patients.
